# OmniMapFree: A unified tool to visualise and explore sequenced genomes

**DOI:** 10.1186/1471-2105-12-447

**Published:** 2011-11-15

**Authors:** John Antoniw, Andrew M Beacham, Thomas K Baldwin, Martin Urban, Jason J Rudd, Kim E Hammond-Kosack

**Affiliations:** 1 Centre for Sustainable Pest and Disease Management, Rothamsted Research, Harpenden, Herts, AL5 2JQ, UK; 2 Centre for Mathematical and Computational Biology, Rothamsted Research, Harpenden, Herts, AL5 2JQ, UK

## Abstract

**• Background:**

Acquiring and exploring whole genome sequence information for a species under investigation is now a routine experimental approach. On most genome browsers, typically, only the DNA sequence, EST support, motif search results, and GO annotations are displayed. However, for many species, a growing volume of additional experimental information is available but this is rarely searchable within the landscape of the entire genome.

**• Results:**

We have developed a generic software which permits users to view a single genome in entirety either within its chromosome or supercontig context within a single window. This software permits the genome to be displayed at any scales and with any features. Different data types and data sets are displayed onto the genome, which have been acquired from other types of studies including classical genetics, forward and reverse genetics, transcriptomics, proteomics and improved annotation from alternative sources. In each display, different types of information can be overlapped, then retrieved in the desired combinations and scales and used in follow up analyses. The displays generated are of publication quality.

**• Conclusions:**

OmniMapFree provides a unified, versatile and easy-to-use software tool for studying a single genome in association with all the other datasets and data types available for the organism.

## Background

In the late 1990s, the first fully sequenced genome of a eukaryotic organism emerged as a result of a huge community effort. The annotated genome of *Saccharomyces cerevisiae *was subsequently published [1] and a comprehensive genome browser has gradually evolved [2,3]. The success of this whole genome sequencing (WGS) project using the Sanger method, paved the way for other model species as well as industrially, agriculturally and medically important species to be nominated for WGS [4]. Within a few years and following the development of several next generation sequencing technologies, the number of eukaryotic species for which complete or near completely sequenced genomes became available steadily rose [5]. Also for the species initially sequenced other strains with different biological properties and closely related species have now been sequenced or nominated for sequencing to provide important clusters of genomic information. In agricultural, environmental and medical research, many species of interest have small to medium sized genomes. For example, free living and pathogenic fungi have genome sizes in the range 12-95 Mb, oomycetes are typically in the range 50-250 Mb, nematodes are in the range 54-169 Mb, whilst insects, are in the range 115-530 Mb [5]. These genomes are ideally suited to the application of next generation sequencing approaches. The rate at which WGS information for multiple species is now being delivered into the public domain is increasing on a monthly basis. Due to the overall reductions in sequencing costs, a high proportion of this new wave of whole genome sequencing (WGS) projects is occurring in academic and industrial establishments and not at the major sequencing centres.

For many eukaryotic species nominated for genomic sequencing there is already a considerable amount of additional biological, biochemical, genetic and molecular information available. Typically, the genome browsers at all the major sequencing centres provide high quality genomic sequence information displays, detailed annotation of this sequence and the results of specific inter-species comparative studies. Usually expressed sequence tag (EST) support is provided to strengthen the gene locus assignments. However, the vast majority of the experimental evidence obtained from forward and reverse genetics experiments, from classical genetic maps for the organism, or from different types of 'omics experiments, i.e. transcriptomics and proteomics, is rarely added to these genome browser or not until they are well advanced. As a consequence early as well as later versions of genome browsers lack this wealth of additional information for the sequenced organism. Immediate access to these other data types alongside the sequenced genome would accelerate the speed of discovery, assist comparative genomic analyses and enhance the development of experimentally testable hypotheses. It is also noticeable that most genome browsers operate within only a very limited range of scales, often doing best at the high magnification ranges, i.e. displaying within a single window a sequence containing < 10 contiguous genes, whilst moving between high and low scales is laborious.

A survey of the other genome map tools currently available indicates that all have pre-set restrictions which severely limit their usefulness when attempting to link additional data types to a newly sequenced and assembled genome. For example, MapMaker focuses on mapping major loci and quantitative trait loci and not on DNA sequence information. The Integrative Genomics Viewer is similar to the browsers available at the major sequencing centres but is web-based. The ENSEMBL browser, GBrowse and Distributed Annotation System (DAS) versions of a species' genome, although providing a wealth of genome and feature annotation when an entire community is actively engaged, tend to be less well populated with genetic and experimentally proven gene function information in their early versions and/or when only a small research community is involved. Within GBrowse there is only the possibility to view the information for single chromosomes/supercontigs which makes interrogation of the entire genome for generic trends and features laborious. Also although these genome browsers are excellent at displaying gene and genomic features, for most only a limited number of datasets are instantly retrievable, for example sequence information, protein domain information. This often limits the types of downstream applications which are possible and hypotheses which can be tested unless a considerable amount of time and effort is spent collating or acquiring additional genome wide data files. These browsers also provide only limited options for the direct generation of publication quality images.

In this article, we describe the development of a new open source software called OmniMapFree which can be used to enrich immediately the information available for an organism with a newly sequenced genome. The aim of this software is to provide a flexible tool for the user to analyse a single genome in combination with an increasing number of different data types/datasets, from other studies on that organism including classical genetics, forward and reverse genetics, transcriptomics, proteomics and improved annotation from alternative sources. OmniMapFree (hereafter simply referred to as OmniMap) does not itself analyse the genome sequence but displays the results of analysis by other software, e.g. blast, blat, tmhmm, signalp and pfam. These results are used to generate data files understood by OmniMap. This can be done using AWK, Python or other scripting languages.

This generic software can be customised to display the genome at multiple scales and with multiple features or to view the genome in entirety within its chromosome context. From each display, information can be retrieved in the desired combinations, and this retrieved information can then be used in follow-up analyses. The displays generated are of publication quality.

### Implementation

The OmniMap software was developed in Delphi for Windows 32 using Embarcadero Delphi XE. The compiled exe file (supplied as Additional file 1) runs on Microsoft ™ Windows XP and Windows Vista operating systems. It also runs on Linux (Red Hat Enterprise Linux 5.2) in the Windows 16/32 bit emulator Wine (1.0.1 and 1.3.13). Other operating systems have not yet been tested. The OmniMap software, updates and pre-configurations can also be freely downloaded from its web site [6].

OmniMap is the generic name for software which can be easily adapted by the user to display and explore maps of different genomes without changing the source code or recompiling the program. In this article, the specific software is called Fgra3Map for the *Fusarium graminearum *genome (version FG3), MgraMap for *Mycosphaerella graminicola*, NcraMap for *Neurospora crassa *and UmayMap for *Ustilago maydis*. But users can rename the exe file and this new name is then displayed on the user interface and within the help file. There is no limitation on the size of genome which can be used in OmniMap but the maximum number of chromosomes/scaffolds is currently set to one thousand.

OmniMap uses tab-delimited text data files and optional FASTA sequence files. The same compiled exe file is used for all genome maps only the data files are different. This makes it easy for users to produce custom genome maps for their favourite organism without needing to change or recompile the Delphi source code. However the source code is freely available from the OmniMapFree web site [6] under the GNU GPL licence. The data files used to generate the OmniMaps for the various species explored were downloaded directly from the sequence provider's websites.

When OmniMap runs the program, checks are done to see if there are sub-folders for *_data, _seqs*, and *_stats *within the folder containing the program's executable file. If these folders do not already exist these are automatically created. These folders which begin with an underscore are special and are not used to make the main menus at the top of the user interface.

### Descriptions of the three special sub-folders

#### _data folder

This folder contains the chromosomes, genes, colours, credits files and optionally any FASTA sequence files of the chromosomes.

The ***chromosomes ***file contains the *id *and *length *of each chromosome or scaffold in the genome with the information for each chromosome on a separate line. The format is id = length e.g. for *Fusarium graminearum *the first line is "1 = 11723881" where "1" is the id of the first chromosome and "11723881" is its length in base pairs. The chromosome ids must be the same as those used in the gene data files so that OmniMap knows on which chromosome each gene is located.

The ***genes ***file is a tab-delimited text file containing a list of the chromosome id, first and last nucleotide positions, strand and gene id of each gene in the genome. Each gene is on a separate line and each of these five fields is separated by a TAB character. There can also be other fields after these, also separated by TABs, containing annotation and other information on the gene. Any blank lines or lines beginning with a "#" character are treated as comments and are ignored.

The ***colors ***file is a list of colour names recognised by OmniMap with each colour on a separate line using the format "clColor = $RGB" where $RGB is the hex Red, Green Blue value of the colour. These are in addition to the 16 standard Windows colours.

The **credits **file is a plain text file containing information specific to the current map e.g. who created it and what source data was used. It is displayed in the map about box under the help menu.

The FASTA file(s) are optional. If the sequence of a chromosome is available it can be provided in this folder and named "*id.fasta*" where id is the chromosome id. If available this information can be used by OmniMap to generate FASTA sequence fragments for any regions selected by the user.

#### _seqs folder

If during an OmniMap session the user saves any selected sequences they are saved in this folder. The selected sequence for each chromosome is saved in a separate file named "id_firstnt-lastnt.fasta" where id is the chromosome id, and the firstnt-lastnt describes the start and end nucleotide position of the sequence in the chromosome.

#### _stats folder

When OmniMap starts it loads in the chromosomes and genes files in the _data folder. The program separates out the genes on each chromosome and saves the genes in each chromosome and their positions in the chromosome in separate files named "id.genes.txt" where id is the chromosome id. It also determines the number of genes on each chromosome and the average number of base pairs per gene. These simple statistics are saved in the stats file in this folder. When the genome of a species is available only as supercontigs or scaffolds, these can also be used to provide the data which forms the basis of the map.

### Displaying genes and other features

Any sub-folders, except those beginning with an underscore, in the same folder as the OmniMap.exe file are used by OmniMap to generate menus on the main-menu bar at the top of the user interface. These menu folders can contain further menu folders and data files. These data files form menu items which when clicked display the genes or features they contain on the map. Users can design the folders and data files they contain and OmniMap will generate a menu structure mirroring the folder structure. The names of the menus and menu items are taken from the folder and file names.

Selecting items on the menu bar causes the relevant genes, ORFs, blast hits, chromosome regions, to be drawn on the chromosomes as coloured blocks each one at a position and size consistent with the feature's actual position and length. The information required to draw each set of genes for each menu item is contained in the data file of the same name in the relevant data folder. The data files are text files which could be produced by the user using a plain text editor, for example, Notepad-but because of the large number of genes in a fungal genome they are usually generated using an AWK, Python, Ruby or Perl script. The OmniMap software just displays features on the chromosomes by using the individual chrom_id, and the start_nt and end_nt of each feature. Therefore this software can in a sense accommodate genomes of any size.

### Data file structures

The data file formats were designed to be as simple and concise as possible. The data files have file extensions of ". **posn**", ".**blast**", ".**expr**", ".**freq**" and ".**graph**" and the way the genes and features are displayed depends on the file extension and data file contents. However all five types of data file are tab-delimited text files. In data files there are three types of lines:

1. Lines beginning with a "#" symbol. The first #-lines contain information about how to draw all features. Other #-lines are either comments or metadata describing the data fields.

2. Blank lines are used to help make the file more human readable and are ignored by the software.

3. All other lines contain actual data in tab-delimited text format. Each line describes the position, size and other information for one gene or feature. In all data files this consists of fields for chromosome id, start position, end position, strand and gene id. Different types of data file also contain other information. It is recommended that the data lines are sorted by chromosome id, and start position so that it is easier to find a gene or feature by position.

## posn data file

This is used to draw genes or features based on their position in the genome (i.e. chromosome id and position on the chromosome). The first #-line contains the colour used to display the genes or regions. Users can put different genes in different posn data files and have them drawn in different colours. The data fields: chromosome id, start position and end position are used to draw the gene.

## blast data file

This is used to display genes or regions of a chromosome hit by a blast search. The first #-line contains the colour used to display the genes or regions. The second #-line gives the E-value cut-off-any data with E-values higher than this are not displayed. The data fields: chromosome id, start position and end position are used the strand and gene id are not but after these are fields for query id and E-value. There must be at least one query id + E-value pair. There can be as many query id + E-value pairs as you want but they should be arranged in order of lowest to highest E-value so that OmniMap only has to check the first E-value to decide whether to display a gene or feature. If the data line represents a region of the chromosome rather than a gene the gene id should be replaced by "seq".

## expr data file

This is used to display genes or features using different colours for example microarray expression data with induced genes coloured red and repressed genes coloured blue. All #-lines are ignored. The data fields: chromosome id, start position and end position are used to draw the gene. The last field on each line is the colour.

## freq data file

This is used to display chromosome regions in different colours. Up to 20 different colours can be used so users can create colour gradients to represent gene density, %GC, or recombination frequency. The first #-line contains a tab-delimited list of colour and cut-off values. The data fields: chromosome id, start position and end position are used to draw the region on the relevant chromosome. The last field on each line is a number value which is used in combination with the colour + cut-off value list to determine the colour displayed.

## graph data file

This is used to display a graph or histogram along a chromosome, e.g. the SNP density using the count of SNPs/50,000 nt. The value of the last field on a data line is used to determine the y-value on the graph.

### Searching for a Gene or Feature

Above the map is an edit box where users can enter a gene id they want to display, then they can select the colour in the colour list box and finally click the "Quick Search" button. OmniMap searches for the gene id in the genes file in the _data folder and if found displays it using the selected colour. The gene can also be redisplayed later using the "Temp\temp" menu. By default the Find Gene ID edit box contains the id of a gene near the middle of the first chromosome.

Several gene ids can be more easily searched for using the "Search\search..." menu. This opens a new window with a list box where the gene ids can be typed, one id per line, or pasted. The display colour can be selected from a colour list box and a name entered in the list name edit box. Clicking the "Search" button causes OmniMap to search for the gene ids in the genes file in the _data folder and if found displays them using the selected colour. The genes can also be redisplayed later using the "Temp" menu in which a new menu item has been created with the user defined name of the list.

The 'exagerate' option found within 'file' menu option can subsequently be selected to widen the width of the displayed genes. This is particularly useful to locate small features/genes when the entire genome is in view. The 'exagerate' option can be switched on and off as and when required.

### The Map

**Figure 1:**
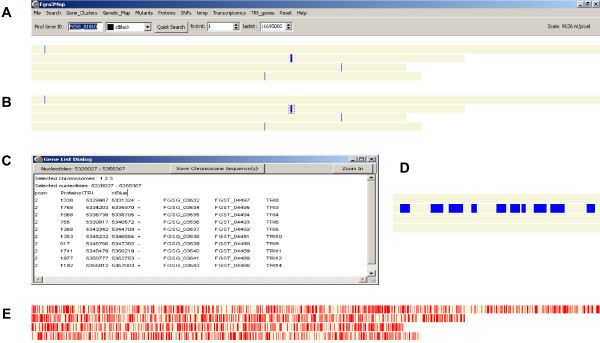
**Screenshots of OmniMap displaying the four *Fusarium graminearum *chromosomes and gene information**. (**a**) *TRI *genes displayed by selecting the *TR*I_genes menu from the top menu bar. The four chromosomes are drawn to scale in grey and the genes are displayed in blue. The species specific OmniMap name, Fgra3Map, is displayed top left. The other menu features shown are described in the main text and Table 1. (**b**) Selection of the main cluster of *TRI *genes in the middle of chromosome 2 using the mouse. The selected region is shown as a dashed rectangle. (**c**) This shows the text data for the *TRI *gene cluster selected in panel b. The third line of the text window: posn TRI_genes\TRI_genes clBlue indicates the data file was of type "posn", its path relative to Fgra3Map.exe was "\TRi_genes\TRI_genes.posn" and the genes are displayed in blue. The following 10 lines give information about each gene. The columns from left to right cover chromosome id, gene length (nt), first nt of gene, last nt of gene, the coding DNA strand, gene id (assigned by BROAD) and the gene annotation [7]. (**d**) A 'zoomed in' display of the chromosome region selected in panel (**b**), revealing the presence of the 10 *TRI *genes (blue boxes). **(e) **Genome wide distribution of the 2,002 *F. graminearum *genes which are considered to be species specific (BLAST E-value cut-off: e^-10^). The data files were produced from the *Fusarium graminearum *Genome Database (FGDB) at the Munich Information Centre for Proteins Sequences (MIPS) version FG3.

The OmniMap program draws the chromosomes/supercontigs/scaffolds on the screen as grey horizontal bars with lengths proportional to their size. The initial view is of the complete genome. When there are too many chromosomes to display on screen at one time (typically n > 30) a vertical scrollbar appears to the right of the map and this can be used to scroll down and up to see all of the genome. When the mouse pointer is moved along a chromosome information about the chromosome identity (ID) and nucleotide position is displayed in the status bar below the map. In Figure 1a the four chromosomes of *F. graminearum *are displayed. For genomes where the chromosomes have yet to be assigned, the OmniMap program can display the assembled scaffolds in decreasing size order or in any other way depending on their order in the chromosomes data file. Where the genome contains unassigned contigs, these can be ordered into a 'pseudo chromosome'. To achieve this, the user concatenates the DNA data placing runs of NNNs between the contigs and displays it as a single unit. This can be added to the bottom of the display (data not shown). The chromosome/supercontigs/scaffolds can be given identical names or numbers to those provided by the sequencing centre/global community or named to fit the individual research group's needs. For some species, the chromosome numbers refer to those given when the first genetic maps were created and therefore do not follow the size order. The genome of *Fusarium graminearum *is of this type [7].

In an OmniMap display of the genome, users can move the mouse to select a region of the map, which is then surrounded by a dotted rectangle. When the mouse button is released the Gene List Dialog appears giving data about any genes or other features displayed in the selected region. This is demonstrated in Figure 1a-d for the *TRI *genes involved in the regulation and synthesis of trichothecene mycotoxins [8-19] which have been selected from the upper menu bar and displayed on the fusarium chromosomes. The *TRI *gene cluster on chromosome 2 was then selected using the mouse (Figure 1b) and appears in the Gene List Dialog Box (Figure 1c). All the data displayed in the dialogue box can be copied and pasted into other applications, for example an Excel spreadsheet. Alternatively if the shift button on the keyboard is held down when the mouse button is released the map zooms in to display this region full screen. The first and last nucleotide positions of the region shown by the map is given in the "firstnt" and "lastnt" spin edit boxes above the map. This process can be repeated to zoom further in and reversed by using the "Reset" "Undo last zoom" or "Zoom fully out" menu at the top of the user interface. Alternatively if the co-ordinates of the region to be examined are known these can be typed into the "firstnt" and "lastnt" spin edit boxes to zoom in. The availability of zoom-in, zoom-out and scrolling enables the user to examine features or groups of features in more detail and at an appropriate scale (Figure 1d).

Each screen map can be saved as a bitmap file using the "File\Save screen map" menu. Alternatively, the genes displayed on the whole genome map can be saved as a file using the "File\Save genome map" menu. These images can then be edited for use in publications or slide presentations.

The Gene List Dialog box also has buttons to zoom into the selected region and, if the chromosome FASTA sequences are available, to extract and save the sequences of the chromosomes in the selected region as FASTA files in the _seqs folder. This makes it easier and more reliable for users to extract sequence specific information for a region or feature of interest.

The features which are displayed by OmniMap for the *F. graminearum *genome are described in Tables 1 and 2. However for each species, the user can decide whether the same types of information need to be displayed or a modified data set is more appropriate. In Figure 1, the screen shot indicates that 12 additional features are available from the upper menu bar as click and search drop downs.

**Table 1:** Description of data file types used by OmniMap and their use

Data file	Type of Data Displayed
posn	Any feature with a position and size on a chromosome or scaffold. For example, genes, primer sequences, markers, homologous regions. Needs chromosome ID, start and end nucleotide positions. The first #-line of the data determines what colour is used to display this region.
blast	Any sequence region identified in a blast search. Needs chromosome ID, start and end nucleotide positions and E-value. All data with E-values less than the E-value cut-off on the 2^nd ^#-line of the data are displayed.
expr	This allows different items in the same data file to be displayed in different colours. For example, with microarray expression data all up-regulated genes could be in red and all down-regulated genes in blue. The colour is determined by the last field on each data line.
freq	This is used to display frequency data. For example, recombination frequency between genetic markers or %GC across a chromosome in a gradient of colours. The value of the last field on a data line is used to determine which of 20 user-defined colours are displayed.
graph	This is used to draw a graph or histogram along a chromosome, for example SNP density. The value of the last field on a data line (e.g. number of SNPs/50,000 nt) is used to determine the y-value.

**Table 2:** Types of features which can be displayed for a single species and the specific resources used to create the OmniMap for *Fusarium graminearum*

Feature	Resource
Genome sequence	BROAD^♦^
Gene space and gene orientation	BROAD
Intergenic space	BROAD
Gene annotation (1)	BROAD
Gene annotation (2)	MIPS^†^
GC content	own analysis
Genetic map	[23]
Single nucleotide polymorphisms	BROAD
Transcriptomic analyses	PLEXdb^‡^
Proteomics analyses	[47]
Gene function studies	www.PHI-base.org[48]
Exons & introns	BLAT
Signal proteins	SignalP
Transmembrane proteins & domains	TMHMM
GPI anchored proteins	Signal P + big-PI [49]
Domain annotation	PFAM, KOG, CDD
Homologous with other known proteins	BLASTP
Secretome	Signal P/WOLFPSORT

^♦^BROAD *Fusarium *Comparative Database [50]

^†^MIPS *Fusarium graminearum *database [51]

^‡^PLEXdb [52]

**Figure 2:**
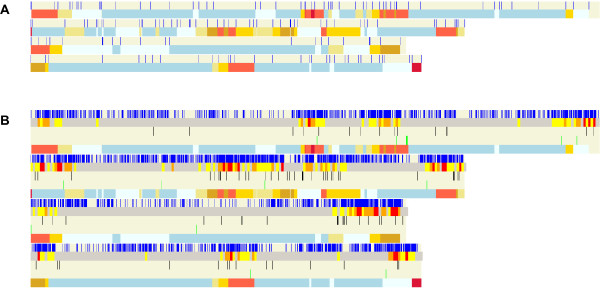
**Screenshots of Fgra3Map linking *Fusarium graminearum *gene information to the genetic map**. (**a**) The position of each molecular marker is shown in blue on each of the four chromosomes. The corresponding frequency of recombination is shown below the marker display for each chromosome using a colour gradient described in the first #-line of the .freq data file i.e. # clBeige 1 clKhaki 2 clGold 3 clGoldenRod 4 clTomato 8 clCrimson. The numbers between the colours are boundary values in cM/27 kb-so beige represents the lowest and crimson the highest recombination frequency. This information was retrieved from [23]. (**b**) The distribution of SNPs in the *Fusarium graminearum *genome compared with genetic recombination frequency and genes coding for putative cell wall degrading enzymes. There are 5 rows shown for each chromosome and each chromosome is separated from the next by a thin, horizontal white line. For each chromosome the top row shows the SNPs as thin, vertical blue lines. This information was retrieved from [23]. The second row shows the SNP density, the third row shows the genes coding for putative cell wall degrading enzymes coloured black, the fourth row shows the genes coding for polyketide synthases and the fifth row shows the genetic recombination frequency (as in Figure 2a).

**Figure 3:**
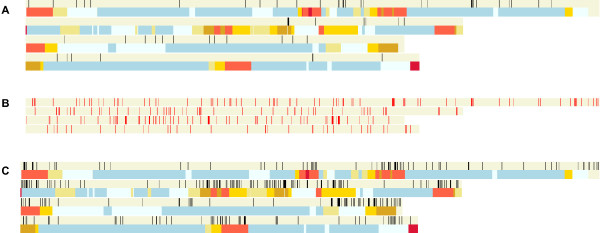
**Screenshots of Fgra3Map linking verified *Fusarium graminearum *gene function information, to gene function predictions and to gene expression information**. (**a**) The distribution of experimentally verified *Fusarium graminearum *pathogenicity and virulence genes. Each gene is displayed in black on the chromosomes. As of April 2011, 61 genes have been published (PHI-base 3.2 and Additional file 2). The second row shows the genetic recombination frequencies. (**b**) The distribution of homologues of pathogenicity and virulence genes revealed through experimentation in a range of plant and animal pathogenic species (PHI-base version 2.1 [44]). The *F. graminearum *homologues were found by BLASTP search using an E-value cut-off of e^-100^. The location of each gene homologue is shown in red on the chromosomes. (**c**) *Fusarium graminearum *genes expressed specifically *in planta *(barley) displayed using the Transcriptomics/Barley *in planta *menu. The genes are displayed in red on the chromosomes. Data sources: see [45] and Plexdb [46] for Fusarium experiments FG1 and FG2.

In addition, the user can search for a single gene locus using the 'quick search' function on the upper menu bar (extreme left). For example throughout Figure 1, the 'Find Gene ID' default display is gene ID fg01810 and the colour selected is black. Alternatively manually generated short lists of gene IDs, or those directly retrieved from Excel spreadsheets or Access databases can be displayed using the main "Search" menu. Each custom gene list can be redisplayed later using the "Temp" menu in which a new menu item has been created with the user-defined name of the list. Alternatively, named files can be created which contain new categories of gene lists and these can be permanently displayed on the upper menu bar. Examples of the latter would be sets of molecular markers used in genetic analyses and differentially expressed genes identified through transcriptomics analyses **(see expr in **Table 1 and Figures 2 and 3). Different colours can be selected to display each gene list separately.

To provide a flexible working environment, the Reset menu can be used to undo the last gene/feature display or undo the last zoom or zoom fully out to display the entire genome.

Similar input files for OmniMap covering genome information have already been generated for the important Ascomycete plant pathogen *Mycosphaerella graminicola *[20] which causes leaf blotch disease of wheat, the model Ascomycete filamentous saprophyte *Neurospora crassa *[21] as well as the model Basidiomycete plant pathogen *Ustilago maydis *[22] which causes smut disease of maize. These maps are freely available from http://www.OmniMapFree.org.

### Identification of genomic locations of the predicted *F. graminearum *species specific genes

For this analysis, required to generate the example display presented in Figure 1e, the NR database was queried on 12^th ^July 2011 with the 13,331 predicted gene sequences in the FG3 version of the genome (BROAD) using BLASTP with a cut-off of E = 1e^-10^.

## Results

The genome of the globally important cereal infecting fungal pathogen *Fusarium graminearum *(teleomorph *Gibberella zeae*) was sequenced in 2003 by the BROAD institute and published [7]. The OmniMap software was developed while exploring this genome, but during development this software was tested on four other sequenced fungal genomes to ensure it retained wide species applicability. These genomes varied in size from 12 Mb to 57 Mb and had from four to 21 chromosomes as well as unassembled DNA. The *F. graminearum *sequence had been aligned to the four genetically assigned chromosomes and these are displayed in OmniMap as four horizontal grey bars (Figure 1). Using the search feature (top left) or the upper menu bar, genes, gene families or genomic features of interest can be displayed. For example, the 10 *TRI *genes involved in the regulation and synthesis of trichothecene mycotoxins [8-19] are displayed on the fusarium chromosomes (Figure 1a) and using the mouse pointer (Figure 1b) the details of the main gene cluster on chromosome 2 can be revealed in the Gene Dialog List displayed in the onscreen pop-up (Figure 1c). Then by using the 'zoom in' function (pop-up, top right), the relative position of each gene as well as gene size and spacing can be displayed (Figure 1d).

### Linking the sequenced genome to the genetic map

A genetic map is available for *Fusarium graminearum*, arising from a sexual cross between the sequenced strain PH-1 (NRRL 31084) and the strain MN00-676 (NRRL 34097) [23]. The F_2 _population comprises 111 single ascospore derived progeny and 235 loci with molecular markers (i.e. dCAPs or CAPs (n = 131), VNTRs (n = 31), AFLP (n = 66) and 7 other markers) have been placed on this map. In Figure 2a the marker set is shown over the four chromosomes (upper bar) whilst below the frequency of recombination in centimorgan is given. As previously noted [7] this display reveals that there is no/little recombination (pale-blue and blue colours) in most areas of the genome. The high recombination regions (gold, goldenred, tomato and crimson colours) tend to be associated with the sub-telomeric regions and a few other internal regions of each chromosome which may represent ancient fusion events resulting from the joining together of smaller chromosomes which are still present in the genomes of other Fusarium species [20,24].

To reveal the gene sequence variation which exists within a single species, in many of the genomic sequencing projects additional isolates of the same species are also sequenced although at a lower overall coverage. For *F. graminearum *approximately x0.4 coverage was provided for a second isolate of USA origin called GZ3639. This revealed the presence of 10, 495 single nucleotide polymorphisms (SNPs) [7]. Using OmniMap this important information can be displayed in graphic format alongside any other genomic, genetic or genetics based data set. For example in Figure 2b, the relationship between SNP occurrence and recombination frequencies has been displayed. By adding the annotated genes list to this display, the SNPs present in each gene can immediately be identified.

When exploring the genome the distribution pattern of the members of expanded gene families can be readily explored in OmniMap. For example, the majority of genes which code for predicted cell wall degrading enzymes or polyketide synthases (PKS) predominantly reside either in regions of either high/very high recombination or high SNPs density (Figure 2b, **rows 3 and 4, respectively**). By using the mouse pointer, it is possible to determine the genes neighbouring each *PKS *gene which potentially reside within each secondary metabolism clusters [25-28] as well as the nucleotide polymorphism present with each gene sequence.

When a genome is sequenced a significant proportion of the genes have no annotation as a result of comparative species analyses or domain searches. Initially therefore, the position of this type of gene in the genome provides the only point of reference. For *F. graminearum*, currently 2,002 of the 13,331 predicted genes (15%) are considered to be species specific (BLASTP with a cut-off of E = 1e^-10^). These genes reside in all regions of the genome (Figure 1e).

### Analysis of the distribution of experimentally verified and putative *Fusarium graminearum *pathogenicity/virulence genes

For many pathogenic species through various forward and reverse genetics experiments, many genes have been shown to contribute to its disease causing ability on one or more hosts. For *Fusarium graminearum *sixty-one genes have been verified to have a role in pathogenicity or virulence (version PHI-base 3.2 and Additional file 2: List of *F. graminearum *virulence genes). These include genes which code for three different MAP kinase signalling cascades, a secreted lipase, glucosylceramide synthase, HMG-CoA reductase, a non-ribosomal peptide synthetase, a siderophore biosynthetic gene, G protein alpha and beta subunits, a novel adenylate-forming enzyme, homoserine O-acetyltransferase, methionine synthase, cystathionine beta-lyase, ubiquinone oxidoreductase, a transducin beta subunit, a putative transcription factor and putative signalling scaffold protein, an F-box protein, acetylglutamate synthase, phosphoribosylamine-glycine ligase, a Ras GTPase, a topoisomerase 1 and a few genes with no assigned biochemical function (eg FSGS_00007). Displaying the entire pathogenicity/virulence gene set using the search function (Figure 3) revealed that these genes are randomly distributed over two of the four *F. graminearum *chromosomes and are located in regions of either no or low recombination. On chromosome 2, three *TRI *genes required for trichothecene mycotoxin production have also been shown to contribute to virulence on wheat. This *TRI *gene cluster exhibits a moderate recombination rate. On chromosome 1, a micro-region containing now experimentally proven virulence genes exists (see below, Beacham, Urban, Freeman, Welham and Hammond-Kosack, unpublished). A recent study of a *F. graminearum *reduced pathogenicity mutant recovered from a forward genetic screen, based on random plasmid insertion, revealed that a 350 kb region containing 146 genes had been lost from the left side of chromosome 1 [29]. The OmniMap software was used to retrieve the sequences present in the wild-type strain and investigate them for the presence of experimentally verified as well as putative pathogenicity and virulence genes [30].

To identify putative pathogenicity and virulence genes in the *F. graminearum *genome, the 348 unique gene sequences present in the pathogen-host interactions database, PHI-base version 2.1 [30,31] was used to search for homologues using BLASTP with a cut-off of E = 1e-^100^. This identified 211 potential homologues and their genomic location is displayed in Figure 3b. This analysis revealed a near random pattern of distribution over each of the four chromosomes, with the exception of a micro-region on chromosome 1 which appears to contain a cluster of pathogenicity and virulence gene homologues [32] and manuscript in preparation). When this information is displayed at the same time as the transcriptomics datasets (see below) it is possible to link immediately gene expression with potential gene function information.

### Analysis of whole genome transcriptomics datasets

In an earlier analysis [7] it was noted that genes revealed to be specifically expressed soon after infection of susceptible barley ears (i.e. 24-96 hours post inoculation) were preferentially distributed to the sub-telomeric regions in areas of high recombination (Figure 3c). Since this initial dataset was published a further twelve microarray experiments have been published and these data are available from the Barleybase (PLEXdb) website [33]. These have been displayed using this software to explore their genomic distribution patterns and to link this with other data types, datasets and bespoke sequence annotation analyses (data not shown).

### Use of OmniMap for the identification of potential sites for genetic variation amongst individuals of the same species

**Figure 4:**
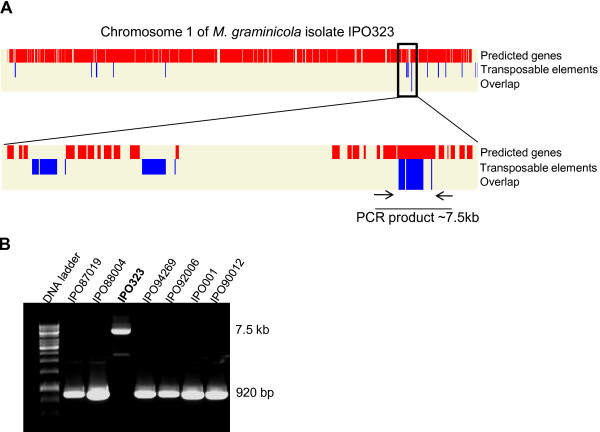
**Screenshot of OmniMap analysis on the presence of transposable elements within open reading frames on chromosome 1 of the *Mycosphaerella graminicola *genome**. The specific map developed for this organism is called MgraMap and was created using the whole genome downloads available from the JGI (Version 2). (**a**) Top bar shows the position of all predicted genes (in red vertical bars). Middle bar shows the position of one transposable element which is identified many times throughout the entire genome sequence (in blue vertical bars). Bottom bar highlights the position of 1 ORF (Gene model Id = e_gw1.1.932.1) which has been disrupted through the integration of the transposon (shown in close-up below with the position of analytical PCR primers). (**b**) Follow up PCR on genomic DNA from seven *M. graminicola *isolates. This analysis confirmed that the open reading frame (ORF) interrupted by transposon insertion in isolate IPO323 was intact (insertion free) in the other six isolates tested. This represents an example where OmniMap can be used to highlight genomic regions which could be the source of genetic variation between individuals of a species.

OmniMap is able to easily identify areas of sequenced genomes which may be variable amongst individuals of the same species and may impart phenotypic variation. For example the sequenced genome of the wheat infecting fungus *Mycosphaerella graminicola *of isolate IPO323 contains a number of transposable elements which occur in numerous copies throughout the 21 chromosomes [20]. One example is provided in Figure 4a for a transposable element which was previously shown to be actively transcribed under particular environmental conditions [34]. Using the OmniMap software to create MgraMap it was possible to identify regions of the genome where transposon integration has occurred within a predicted open reading frame (ORF) (Figure 4a). It was then possible to determine whether this ORF was disrupted in other *M. graminicola *isolates by performing follow-up Polymerase Chain Reaction (PCR) analysis on genomic DNA spanning this region (Figure 4b). For this particular example it was shown that the other six *M. graminicola *isolates tested lacked the transposon insertion in this particular ORF in Chromosome 1 of the genome, and thus likely possesses a functional copy of this gene.

### Additional features of the OmniMap software

The software was designed to be simple to understand and use but flexible and powerful enough to be useful in fundamental research. This was achieved by providing a compiled executable file which can be customised for different genomes without recompiling but by using different data files. Also the data is stored as simple tab-delimited text files so no complex databases are involved. One major difference between this genome browser and any other is the ability to begin each analysis with a display of the entire genome. This greatly increases the researcher's spatial awareness of the entire genome landscape during each analysis.

The software and data for a genome are kept in a single directory tree with the executable located in the top folder. The data files are organised in folders and sub-folders. The software generates its main menu system and sub-menus from the directory structure of the data folders and sub-folders giving the user the ability to modify the user interface in a simple, familiar manner.

This design also makes it easy for users to share genome maps-simply by compressing the top folder and all sub-folders and files into a zip or tarball file, and transferring it to another computer where it can be uncompressed and used. Pre-designed genome maps for a number of organisms are freely available on the OmniMapFree web site [6]. This site also acts as a repository for user's genome maps.

## Discussion

The OmniMapFree software provides individual users and/or research teams with a versatile and flexible tool that can be further custom developed to suit many different types of genome analyses. The potential to display a sequenced genome from whole chromosomes/supercontigs, through a continuum of scales down to a single gene or nucleotide within a sequence of interest within a few seconds also means that many different types of hypotheses can be tested simultaneously. When data sets become available from websites which are unavailable from the primary genome sequence browser, or have recently become available in the peer reviewed literature, or from within the research group, these can be either added individually to the upper menu bar as permanent features or as named temporary files. Although OmniMapFree does not itself analyse the genome sequence, it can display the results of analysis by other software, e.g. blast, blat, tmhmm, signalp, pfam etc and thereby enrich the annotation information available.

This software has so far been tested on the sequenced genomes of five different plant pathogenic fungi, namely *Fusarium graminearum *(Versions FG1 and FG3), *F. oxysporum *f. sp. *lycopersici, F. verticillioides, Mycosphaerella graminicola *(Versions MG1 and MG2), *Ustilago maydis *and the model organism *Neurospora crassa*. This has ensured that this software has retained generic applicability and has been used on genomes containing up to 65 Mb (*Fol*). Thus the software is readily applicable to the numerous new pest and pathogen genome sequencing projects as well as for projects involving the resequencing of additional isolates and strains of a single species which possess different biological properties.

When the genome of an organism is sequenced and the predicted gene call compared by BLAST/BLAT analyses to the sequences in NCBI, Prosite and other databases, many genes remain completely non-annotated. For example, for the recently sequenced obligate biotrophic plant pathogens *Blumeria graminis *f. sp. *hordei *which cause powdery mildew disease on barley, 37.4% of the 6121 genes now predicted have no annotation arising from a BLAST analysis [35] (Spanu, Burgis and Butcher, pers comm.). For the recently sequenced plant insect pest, the green peach aphid *Acyrthosiphon pisum *with a genome size of 517 Mb and with 32,000+ predicted ORFs, the number of genes classified as orphans was 20% [36]. When genome sequence information is placed in OmniMap directly in association with other types of information arising from the organism, for example, genetic mapping studies, transcriptomics and/or proteomics analyses, some spatial information is immediately available for many of these previously non-annotated genes and can be incorporated into follow up analyses. OmniMap is particularly effective when displaying the results of multi-species comparative analyses to reveal the chromosome locations of species-specific gene sequences (eg Figure 1e), a category which often contains a high percentage of otherwise non-annotated genes.

Forward genetics experiments are frequently used to reveal in an unbiased way novel genes required for the disease causing ability of a pathogen towards its plant and/or animal hosts. Although most of the transformants recovered by this experimental approach possess single plasmid, T-DNA or transposon insertion events, sometime more complex re-arrangements occur. Recently, the OmniMap software in combination with a transcriptomics analysis was used to help to fully characterise a *F. graminearum *reduced pathogenicity mutant which had lost at least 146 genes from one end of chromosome 1 [29]. For biologists, geneticists and molecular biologists with minimal training in bioinformatics, the availability of an OmniMap version of the sequenced genome of their experimental organism provides them with considerably greater flexibility in the interpretation of experimental results and the design of follow-up experiments.

For some sequenced organisms detailed genetic maps are also available and involve the sequenced strain. By positioning the sequences derived from the molecular markers on the genetic map onto the genomic sequence alongside the extent of recombination identified within each genetic interval it is possible to use the OmniMap software to define the gene sets and gene family members residing within regions of low, medium or high recombination. This may give some indication of the likelihood of extent of sequence variation that may be exhibited if multiple isolates were explored for sequence variation in this genetic interval. For some projects and downstream applications, for example, genotyping an isolate collection, a high degree of sequence variation is desirable. Whereas for other applications, for example, identifying novel targets for pest and pathogen controls or for diagnostic purposes the identification of genes present only as monomorphic invariant sequences in areas of low recombination in the genome is highly desirable.

The loss of whole small chromosomes may occur in some species during sexual recombination, causing some of the progeny to inherit less than the full chromosome complement. Using OmniMap the types of genes lost during single and multiple small chromosome reduction events can be readily assessed and this information used in follow up bioinformatics analyses. OmniMap has recently been used for this purpose when studying the genome of the wheat specific fungal pathogen *Mycosphaerella graminicola *(anamorph *Septoria tritici*), [20,37-39]. The genome contains thirteen core chromosomes and eight dispensable smaller chromosomes (the dispensome). Progeny from sexual crosses can lose from one to eight of the chromosomes from the dispensome but still retain full pathogenicity towards wheat [39]. Also the OmniMap display of this species has revealed that the predicted gene density on the eight dispensable chromosomes is considerable less than the gene density on the thirteen core chromosomes [20].

When the data is retrieved from an OmniMap display showing multiple features, the selected data subsets can be dropped into Excel spreadsheets for immediate follow-up analysis. There is no visible code within the retrieved data. Similarly, data sets generated manually as lists, or obtained as lists from other sources, can be dropped into the search function and the file appropriately named prior to display. These position files can then be moved from the temp folder into any of main folders. This obviates the need to use a scripting language to generate the correctly formatted new position data files. Therefore once the basic OmniMap version of the genome is available, the researcher can add numerous new files of features without requiring further bioinformatics assistance. This is particularly desirable when testing *in silico*, specific hypotheses.

The ability for the OmniMap software to display the entire genome as either chromosomes or all the scaffolds available in a single screen view means that this software can be used to test hypothesis where a global view of the genome is required. For example, in a recent *Aspergillus fumigatus *study, the *in host *expressed transcriptome was found to be sub-telomerically localised [40]. For genomes where detailed genetic recombination maps are available this software feature will greatly facilitates the genome wide integration of data types. Also in next generation sequencing projects, where multiple strains of the same species are sequenced because each possesses different biological properties, the immediate identification of the position of all single nucleotide polymorphisms (SNP) on a full genome scale in a single view could be highly informative [41].

In the late 1990s, full genome sequence information was available for just a handful of eukaryotic organisms. Since then various national and international sequencing initiatives have provided genome sequence information for representative species across the tree of life and subsequently for the taxonomical 'in-fillers' species. Although the primary sequence providers in the public domain such as the BROAD and JGI in the USA and Sanger Centre and the EBI in the UK provide excellent genome browsers for single organisms or sometimes for a few closely related species (for example the Fusaria at the BROAD), their focus is the display of high quality genomic sequence information, detailed annotation of this sequence and for inter-species comparative studies. Usually expressed sequence tag (EST) support is provided to strengthen the gene locus assignments and only occasionally is this information presented in a manner suitable for biological purposes, for example, the display of separated EST libraries on the *M. graminicola *genome browser at the JGI. Typically new releases of a genome only occur on a yearly basis for the first few years and then stop. With the arrival of the new lower cost second generation sequencing methods, such as 454 and Solid, an increasing amount of genome and transcriptome sequence information is become available in the public domain from smaller sequencing centres and from University and Institute departments. It is highly unlikely that all this new sequence information will be captured alongside the original reference genome for a given species. For some large species groups, the EBI in Cambridge, UK, have developed the ENSEMBL browser to collate different data types and interlink species. These species groups include ENSEMBL vertebrates, other Metazoa, Plants and Fungi and Protists, Bacteria and Achaea [42]. But it often takes time to have new data entered and the display format is relatively fixed. The OmniMap software described in this article we consider is of value to individual researchers and researcher teams where in addition to a sequenced genome many different data types are becoming rapidly available for a sequenced organism from a variety of external sources as well as from their own research. This software has been placed in the open domain and so can be further developed for additional purposes.

## Conclusions

OmniMapFree displays a linear map representing a single genome, showing each chromosome/scaffold to scale as a coloured bar. Numerous features of the user's choice can then be displayed either singly or in combination on the chromosomes as coloured regions or vertical lines. The user can capture regions of interest using the mouse pointer and the full information for this region is displayed in an onscreen popup called the Gene List Dialog. Alternatively, the user can zoom into regions of interest for a more detailed view. This software enables the user to examine the relative positions of different features on the chromosomes and to customise their study. An 'undo button' permits the analysis to be further refined while the analysis is in progress. There are also two types of instant query tool and the results can be saved into files which can be re-displayed. At any time new data sets and data types can be added to the map and immediately displayed without the use of a scripting language. All the information retrieved in each 'onscreen popup' can be immediately downloaded into other analysis applications or spreadsheets to generate tables for publication. Each OmniMap display can also be published directly [29,34,38,43]. This software will be particularly useful to research groups where a wealth of additional information is already available for the newly sequenced organism.

## Availability and requirements

**Project name: **OmniMapFree

**Project home page: **http://www.omnimapfree.org/

**Operating system(s): **Windows XP, Windows Vista

**Programming language: **Delphi

**Other requirements: **none

**License: **GNU GPL

**Restrictions to use by non-academics: **none

## List of abbreviations used

DNA: deoxynucleic acid; EST: expressed sequence tag; GC: guanine cytosine; GO: gene ontology; ID: identity; Mb: megabases; nt: nucleotide; ORF: open reading frame; PCR: polymerase chain reaction; PKS: polyketide synthases; SNP: single nucleotide polymorphism; *TRI: *trichothecene; WGS: whole genome sequencing.

## Authors' contributions

KHK provided biological insight, originated the concept, designed the software requirements, and drafted the majority of the manuscript. JA was responsible for developing all of the software content, helped draft the manuscript and developed the accompanying website. AB, TB, MU and JJR tested alpha and beta versions of the software, made suggestions for further improvements and helped draft the manuscript. In addition JJR generated the data presented in Figure 4. All authors read and approved the final manuscript.

## Authors' information

JA is a computational biologist with a special interest in the analysis of fungal and viral genomes. AB is a molecular biologist with a special interest in signalling in plant and microbes. TB is a molecular biologist with a special interest in plant infecting pathogens. MU is a molecular geneticist with a special interest in the pathogenicity requirements of plant infecting fungi. JR is a biochemist with a special interest in the pathogenicity of plant infecting fungi and the mechanisms underlying host defence.

KHK is a molecular geneticist with a special interest in the pathogenicity of plant infecting fungi and the mechanisms underlying host defence.

## Supplementary Material

Additional file 1 **OmniMap software**. A ZIP file containing the Windows executable and the OmniMap source code. Click here for file

Additional file 2 **The verified *Fusarium graminearum *pathogenicity/virulence genes**. An RTF file listing 61 *F. graminearum *genes contributing to virulence on many host plant species. Click here for file
