# Supplementary material for: OmniMapFree: A unified tool to visualise and explore sequenced genomes
Source: BMC Bioinformatics. 2011 Nov 15;12:447. doi: 10.1186/1471-2105-12-447 (PMC3251307; doi:10.1186/1471-2105-12-447)
Supplement: Additional file 2 — The verified Fusarium graminearum pathogenicity/virulence genes. An RTF file listing 61 F. graminearum genes contributing to virulence on many host plant species. [file 1471-2105-12-447-S2.RTF]

File 2: Verified Fusarium graminearum pathogenicity / virulence genes. Where the original publication has been cited in the PHI-base database (Version3.2), only the PH-base ID is given (www.phibase.org).


FGSG ID♦	Gene name (alias)	Protein function	Cereal hosts ‡	Phenotype †‡	Chromosome location	Reference / PHI-base ID	
Cellular communication / Signal transduction mechanism					
FGSG_01665	FSR1	Putative signalling scaffold protein	B, M	red_vir	1	PHI:731	
FGSG_00332	FTL1 (TBL1)	S. cerevisiae SIF2 (Transducin beta-subunit)	W	non_path	1	PHI:446; {Ding, 2009 #3129}(Ding et al., 2009)
	
FGSG_09614	GPA2	Guanine nucleotide-binding protein alpha-3 subunit	B	red_vir	4	(Yu et al., 2008)	
FGSG_04104	GPB1	Guanine nucleotide-binding protein beta subunit	B	red_vir	2	(Yu et al., 2008)	
FGSG_09612	HOG1/OS2	S. cerevisiae HOG1
(Osmotic stress MAPK)	W	red_vir	4	(Ochiai et al., 2007; Oide et al., 2010)	
FGSG_06385	MAP1(GPMK1)	S.cerevisiae KSS1/FUS3 (mating/filamentation MAPK)	W	red_vir	3	PHI:309	
FGSG_10313	MGV1	S. cerevisiae SLT2
(Cell integrity MAPK) 	W	red_vir	1	PHI:266	
FGSG_09908	PKAR	Protein kinase A regulatory subunit	W	red_vir	1	(Beacham , 2011)	
FGSG_10114	RAS2	Ras GTPase	W, M	red_vir	1	PHI:861	
FGSG_09897	SNF1	Sucrose nonfermenting protein kinase	B, W	red_vir	1	(Lee et al., 2009b) Urban and Hammond-Kosack, unpubl.	
FGSG_05484	STE11	MAPKKK; hypersensitive to MsDEF1	W	red_vir	3	PHI:1016	
FGSG_09903	STE7	MAPKK; hypersensitive to MsDEF1	W	red_vir	1	PHI:1004	
FGSG_04982	TEP1	Tensin-like phosphatase 1
Phosphatidylinositol-3 kinase signalling	WC	red_vir	3	(Zhang et al., 2010)	
Metabolism					
FGSG_02506	ADE5	Phosphoribosylamine-glycine ligase	B	red_vir	1	PHI:744	
FGSG_01939	ARG2	Acetylglutamate synthase	B	red_vir	1	PHI:743	
FGSG_01932	CBL1	Cystathionine beta-lyase	W, M	red_vir	1	PHI:443	
FGSG_05906	FGL1	Secreted lipase	W, M	red_vir	3	PHI:432	
FGSG_05955	GCS1	Glycosylceramide synthase (sphingolipid biosynthesis)	W, M	red_vir	3	PHI:1002	
FGSG_05658	GzmetE	Homoserine O-acetyltransferase	B, M	red_vir	3	PHI:355	
FGSG_09197	HMR1	3-hydroxy-3-methylglutaryl-coenzyme A reductase  involved in isoprenoid biosynthesis	W	red_vir	4	PHI:1006	
FGSG_09896/ FGSG_00176	ICL1 MCL1	Double mutant of isocitrate and methylisocitrate lyase	W, B	red_vir	1/1	(Lee et al., 2009a)	
FGSG_10825	MSY1	Methionine synthase	W, M	red_vir	3	PHI:442	
FGSG_05593	MT2 	Sphingolipid C-9- methyltransferase	W	red_vir	3	(Ramamoorthy et al., 2009)	
FGSG_09895	NTH1	Neutral trehalase	W	red_vir	1	(Beacham, 2011)	
FGSG_05371	SID1	Siderophore biosynthetic gene	W	red_vir	3	PHI:1010	
FGSG_02549	Transposon mutant	Putative phosphoglycerate mutase family	W	non_path	1	(Dufresne et al., 2008)	
Energy					
FGSG_12857	ACL1	ATP citrate lyase	W	non_path	3	(Son et al., 2011)	
FGSG_060393	ACL2	ATP citrate lyase	W	non_path	3	(Son et al., 2011)	
FGSG_00376	NOS1	NADH:Ubiquinone oxidoreductase	W, M	red_vir	1	PHI:445	
Protein fate					
FGSG_10740
	ATG8	Autophagic death protein	W, B	red_vir	3	(Ding et al., 2007)	
FGSG_02095	FBP1	F-box protein involved in ubiquitin-mediated degradation	B	red_vir	1	PHI:733	
Biogenesis of cellular components					
FGSG_12039	CHS5	Myosin-motor like chitinase	B	red_vir	1	(Kim et al., 2009)	
FGSG_12039	CHS7	Myosin-motor like chitinase	B	red_vir	1	(Kim et al., 2009)	
Interaction with the environment 					
FGSG_12970	PAC-Cc	Cys2His2 zinc finger transcription repressor	W	red_vir	4		
FGSG_01974	none designated	Similar to HET-C2 glycolipid transfer protein	W	red_vir	1	(Dufresne et al., 2008)	
FGSG_11955	VE1	Light-responding activator velvet1	W	non_path	1	J. Merhej and C. Barreau, pers. com.	
 Cellular transport, transport facilities and transport routes					
FGSG_00950	 SYN1	SNARE protein (transport docking and vesicle fusion)	B	red_vir	4	(Hong et al., 2010)	
FGSG_09928	SYN2	SNARE protein (transport docking and vesicle fusion)	B	red_vir	1	(Hong et al., 2010)	
FGSG_00416	none designated	Putative major facilitator superfamily	W	red_vir	1	(Dufresne et al., 2008)	
Cell cycle 	 		 		
FGSG_04355	CID1	Cyclin-C-like gene requried for infection and DON production	W, M	red_vir	2	(Zhou et al., 2010)	
Transcription and DNA modification					
FGSG_01353	HDF1	S. cerevisiae HOS2
(Class II histone deacetylase)	W, M	red_vir	1	(Li et al., 2011)	
FGSG_04324 	HDF2	S. cerevisiae HDA1
(Class II histone deacetylase)	W,M	red_vir	2	(Li et al., 2011)	
FGSG_10129	STUA	APSES transcription factor	W	non_path	1	(Lysøe et al., 2011)	
FGSG_06874	TOP1	Topoisomerase 1	W	red_vir	4	(Baldwin et al., 2010b)	
FGSG_10057	none designated	Putative transcription factor (Zn(II)2Cys6 domain)	W	non_path	1	(Dufresne et al., 2008)	
FGSG_01555	ZIF1	B-ZIP transcription factor	W, M	red_vir	1	PHI:444; (Wang et al., 2011)	
Cell rescue, defense and virulence					
daf10	146 genes deleted from end of chromosome 1 incl. FGSG_00071 (tri1)	W	red_vir	1	(Baldwin et al., 2010a)	
FGSG_00007	FGSG_00007	Cytochrom P450 monooxygenase (DON biosynthesis)	W	inc_vir	1	(Gardiner et al., 2009)	
FGSG_10397	FGSG_10397	Unknown function	W	inc_vir	1	(Gardiner et al., 2009)	
FGSG_03747	NPS6	Non-ribosomal peptide synthetase for biosynthesis of extracellular siderophores	W	red_vir	2	PHI:1007	
FGSG_04111	PTC1	Type 2C protein phosphatase	WC	red_vir	2	(Jiang et al., 2010)	
FGSG_03538	TRI10	Regulatory protein	W	red_vir	2	(Seong et al., 2009)	
FGSG_03543	TRI14	Putative trichodiene biosynthesis gene	W, M	red_vir	2	PHI:525	
FGSG_03537	TRI5	Trichodiene synthase	W, M	red_vir	2	PHI:44	
FGSG_03536	TRI6	Transcription factor	W	red_vir	2	PHI:439	
Unclassified protein					
FGSG_06631	CPS1	Adenylate-forming enzyme	W	red_vir	4	PHI:443	
FGSG_09907	FCV1	Conserved hypothetical protein	W	red_vir	1	(Beacham, 2011)	
FGSG_06680	MES1	Role in cell-surface organisation	W	red_vir	4	PHI:1078	
FGSG_02077	none designated	Conserved hypothetical protein	W	non_path	1	(Dufresne et al., 2008)	
FGSG_12019	none designated	Hypothetical protein	W	red_vir	1	(Dufresne et al., 2008)	
FGSG_12753	none designated	Hypothetical protein	W	red_vir	3	(Dufresne et al., 2008)	
Abbreviations: ATP (Adenosine triphosphate), APSES (100 amino acid protein domain), GTP (Guanosine triphosphate), MAPK (Mitogen-activate protein kinase), MAPKKK (Mitogen-activated protein kinase kinase kinase)


♦ FGSG ID (F. graminearum locus identifier) was taken from the F. graminearum genome version 3.0 (Broad and MIPs) 
‡ Cereal hosts tested are wheat spikes (W), barley spikes, wheat coleoptiles (WC), maize (M). 
†‡ Abbreviations:   non_path – non-pathogenic; red_vir – reduced virulence, inc_vir – increased virulence 


References for Supplementary Table 1 

Baldwin, T.K., Gaffoor, I., Antoniw, J., Andries, C., Guenther, J., Urban, M., Hallen-Adams, H.E., Pitkin, J., Hammond-Kosack, K.E., and Trail, F. (2010a). A partial chromosomal deletion caused by random plasmid integration resulted in a reduced virulence phenotype in Fusarium graminearum. Molecular Plant-Microbe Interactions 23, 1083-1096.
Baldwin, T.K., Urban, M., Brown, N., and Hammond-Kosack, K.E. (2010b). A role for topoisomerase I in Fusarium graminearum and F. culmorum pathogenesis and sporulation. Molecular Plant-Microbe Interactions 23, 566-577.
Beacham, A. (2011). Pathogenicity determinants of Fusarium graminearum on wheat ears. In Plant Pathogen and Microbiology (Exceter, Exceter University).
Ding, S.L., Mehrabi, R., Koten, C., Kang, Z.S., Wei, Y.D., Seong, K.Y., Kistler, H.C., and Xu, J.R. (2009). Transducin Beta-Like Gene FTL1 Is Essential for Pathogenesis in Fusarium graminearum. Eukaryotic Cell 8, 867-876.
Ding, S.L., Shou, X., Kistler, H.C., and Xu, J. (2007). FMK1 regulates the expression of 333 genes in Fusarium graminearum [abstract]. Paper presented at: National Fusarium Head Blight Forum Proceedings.
Dufresne, M., van der Lee, T., Ben M'barek, S., Xu, X., Zhang, X., Liu, T., Waalwijk, C., Zhang, W., Kema, G.H., and Daboussi, M.J. (2008). Transposon-tagging identifies novel pathogenicity genes in Fusarium graminearum. Fungal Genet Biol 45, 1552-1561.
Gardiner, D.M., Kazan, K., and Manners, J.M. (2009). Novel genes of Fusarium graminearum that negatively regulate deoxynivalenol production and virulence. Molecular Plant-Microbe Interactions 22, 1588-1600.
Hong, S.Y., So, J., Lee, J., Min, K., Son, H., Park, C., Yun, S.H., and Lee, Y.W. (2010). Functional analyses of two syntaxin-like SNARE genes, GzSYN1 and GzSYN2, in the ascomycete Gibberella zeae. Fungal Genet Biol 47, 364-372.
Jiang, L.H., Yang, J.R., Fan, F.Y., Zhang, D.J., and Wang, X.L. (2010). The Type 2C protein phosphatase FgPtc1p of the plant fungal pathogen Fusarium graminearum is involved in lithium toxicity and virulence. Molecular Plant Pathology 11, 277-282.
Kim, J.E., Lee, H.J., Lee, J., Kim, K.W., Yun, S.H., Shim, W.B., and Lee, Y.W. (2009). Gibberella zeae chitin synthase genes, GzCHS5 and GzCHS7, are required for hyphal growth, perithecia formation, and pathogenicity. Current Genetics 55, 449-459.
Lee, S.H., Han, Y.K., Yun, S.H., and Lee, Y.W. (2009a). Roles of the glyoxylate and methylcitrate cycles in sexual development and virulence in the cereal pathogen Gibberella zeae. Eukaryot Cell 8, 1155-1164.
Lee, S.H., Lee, J., Lee, S., Park, E.H., Kim, K.W., Kim, M.D., Yun, S.H., and Lee, Y.W. (2009b). GzSNF1 Is Required for Normal Sexual and Asexual Development in the Ascomycete Gibberella zeae. Eukaryotic Cell 8, 116-127.
Li, Y., Wang, C., Liu, W., Wang, G., Kang, Z., Kistler, H.C., and Xu, J.-R. (2011). The HDF1 Histone Deacetylase Gene Is Important for Conidiation, Sexual Reproduction, and Pathogenesis in Fusarium graminearum. Molecular Plant-Microbe Interactions 24, 487-496.
Lysøe, E., Pasquali, M., Breakspear, A., and Kistler, H.C. (2011). The Transcription Factor FgStuAp Influences Spore Development, Pathogenicity, and Secondary Metabolism in Fusarium graminearum. Molecular Plant-Microbe Interactions 24, 54-67.
Ochiai, N., Tokai, T., Nishiuchi, T., Takahashi-Ando, N., Fujimura, M., and Kimura, M. (2007). Involvement of the osmosensor histidine kinase and osmotic stress-activated protein kinases in the regulation of secondary metabolism in Fusarium graminearum. Biochem Biophys Res Commun 363, 639-644.
Oide, S., Liu, J.Y., Yun, S.H., Wu, D.L., Michev, A., Choi, M.Y., Horwitz, B.A., and Turgeon, B.G. (2010). Histidine kinase two-component response regulator proteins regulate reproductive development, virulence, and stressr esponses of the fungal cereal pathogens Cochliobolus heterostrophus and Gibberella zeae. Eukaryotic Cell 9, 1867-1880.
Ramamoorthy, V., Cahoon, E.B., Thokala, M., Kaur, J., Li, J., and Shah, D.M. (2009). Sphingolipid C-9 methyltransferases are important for growth and virulence but not for sensitivity to antifungal plant defensins in Fusarium graminearum. Eukaryotic Cell 8, 217-229.
Seong, K.-Y., Pasquali, M., Zhou, X., Song, J., Karen, H., McCormick, S., Dong, Y., Xu, J.-R., and Kistler, H.C. (2009). Global gene regulation by Fusarium transcription factors Tri6 and Tri10 reveals adaptations for toxin biosynthesis. Molecular Microbiology 9999.
Son, H., Lee, J., Park, A.R., and Lee, Y.W. (2011). ATP citrate lyase is required for normal sexual and asxexual development in Gibberella zeae. Fung Genet Biol in press.
Wang, Y., Liu, W., Hou, Z., Wang, C., Zhou, X., Jonkers, W., Ding, S., Kistler, H.C., and Xu, J.-R. (2011). A novel transcriptional factor important for pathogenesis and ascosporogenesis in Fusarium graminearum. Molecular Plant-Microbe Interactions 24, 118-128.
Yu, H.Y., Seo, J.A., Kim, J.E., Han, K.H., Shim, W.B., Yun, S.H., and Lee, Y.W. (2008). Functional analyses of heterotrimeric G protein G alpha and G beta subunits in Gibberella zeae. Microbiology 154, 392-401.
Zhang, D., Fan, F., Yang, J., Wang, X., Qiu, D., and Jiang, L. (2010). FgTep1p is linked to the phosphatidylinositol-3 kinase signalling pathway and plays a role in the virulence of Fusarium graminearum on wheat. Molecular Plant Pathology 11, 495-502.
Zhou, X., Heyer, C., Choi, Y.E., Mehrabi, R., and Xu, J.R. (2010). The CID1 cyclin C-like gene is important for plant infection in Fusarium graminearum. Fungal Genet Biol 47, 143-151.
